# Hsa_circ_0007292 promotes chondrocyte injury in osteoarthritis via targeting the miR-1179/HMGB1 axis

**DOI:** 10.1186/s13018-023-04026-7

**Published:** 2023-07-29

**Authors:** Zhiping Lin, Peng Li, Yangyang Tang, Hongchang Tan, Lianxiang Luo

**Affiliations:** 1 Orthopedic Center, The Affiliated Hospital of Guangdong Medical University, Zhanjiang, 524001 China; 2 Stem Cell Research and Cellular Therapy Center, The Affiliated Hospital of Guangdong Medical University, Zhanjiang, 524001 China; 3 The Marine Biomedical Research Institute, Guangdong Medical University, No.2, Wenming East Road, Zhanjiang, 524023 China

**Keywords:** Hsa_circ_0007292, Osteoarthritis, Chondrocyte, miR-1179, HMGB1

## Abstract

**Background:**

Circular RNAs (circRNAs) have been demonstrated to participate in the progression of osteoarthritis (OA). This study aimed to investigate the role and molecular mechanism of hsa_circ_0007292 in OA.

**Methods:**

Hsa_circ_0007292 was identified by analyzing a circRNA microarray from the Gene Expression Omnibus (GEO) database, and its expression was detected by real-time PCR in OA cartilage tissues and interleukin (IL)-1β-induced two human chondrocytes (CHON-001 and C28/I2), the OA cell models. The effects of hsa_circ_0007292 knockdown and miR-1179 overexpression on IL-1β-induced chondrocyte injury were examined by CCK-8, BrdU, flow cytometry, ELISA, and western blot. RNA pull-down assay and dual-luciferase reporter gene assay were used to analyze the interaction between hsa_circ_0007292 and miR-1179. Rescue experiments were carried out to determine the correlations among hsa_circ_0007292, miR-1179 and high mobility group box-1 (HMGB1).

**Results:**

Hsa_circ_0007292 expression was upregulated in OA tissues and IL-1β-induced chondrocytes. Both downregulation of hsa_circ_0007292 and miR-1179 overexpression increased the proliferation and Aggrecan expression, suppressed apoptosis, matrix catabolic enzyme MMP13 expression and inflammatory factor (TNF‐α, IL‐6, and IL‐8) levels. There was a negative correlation between hsa_circ_0007292 and miR-1179, and a positive correlation between hsa_circ_0007292 and HMGB1 in OA tissues. The mechanistic study showed that hsa_circ_0007292 prevented HMGB1 downregulation by sponging miR-1179. Upregulation of HMGB1 could reverse the influence of hsa_circ_0007292 downregulation on IL-1β-induced chondrocyte injury.

**Conclusions:**

Downregulation of hsa_circ_0007292 relieved apoptosis, extracellular matrix degradation and inflammatory response in OA via the miR-1179/HMGB1 axis, suggesting that hsa_circ_0007292 might be a potential therapeutic target for OA treatment.

## Introduction

Osteoarthritis (OA), one of the most types of arthritis, is a chronic degenerative joint disorder mainly characterized by progressive erosion of articular cartilage [1]. This disease is one of the most common causes of disability that largely reduces the quality of life in patients and leads to huge healthcare and socioeconomic burdens [2]. It is estimated that over 22% of adults > 40-year-old have knee OA, and OA affects more than 500 million individuals worldwide in 2021 [2, 3]. What’ worse, the global incidence of OA is increasing, it is predicted that about 25% of adults will suffer from OA by 2030 [4]. Currently, the clinical treatments of OA are only symptomatic therapy like improving joint pain symptoms, rather than declining the disease progression, which is partially due to the lack of comprehensive understanding to the initiation and development of this disease [5, 6]. Hence, it is of great importance to find the factors inducing OA and the underlying mechanisms [2].

Articular cartilage is a highly specialized connective tissue with only one cell type (chondrocytes) and extracellular matrix (ECM) produced by chondrocytes [7]. The balance between synthesis and degradation of ECM molecules, such as type-II collagen and aggrecan, is tightly regulated in chondrocytes of normal adult cartilage, providing the integrity of structure and function of cartilage [8]. In OA, the generation of ECM is repressed, while the matrix-degrading enzymes are over-produced, leading to the excessive degradation of ECM [9]. The pro-inflammatory cytokines, such as interleukin (IL)-1β, tumor necrosis factor (TNF)-α, and IL-6 secreted by inflamed OA synovium and damaged cartilage can also stimulate chondrocyte catabolism and apoptosis [7, 10, 11]. The apoptosis of chondrocytes is a vital process in the initiation and progression of OA [7, 12].

Circular RNAs (CircRNAs) are single-stranded and covalently closed noncoding RNAs without a free 3’ or 5’ end, which makes them more stable than linear mRNAs. They are highly conserved and tissue-specifically distributed in eukaryotic cells [13]. Many circRNAs function as decoys or sponges of microRNAs (miRNAs), leading to the repression of miRNA function [14]. Studies have demonstrated that circRNAs were involved in many biological processes, including proliferation, apoptosis and inflammatory responses, and associated with the occurrence of many human diseases, such as cancers, neurological dysfunction, atherosclerosis and diabetes [15–18]. In recent years, circRNAs have been found to be differentially expressed in OA patients and participate in the development of OA [19].

Hsa_circ_0007292 is a circRNA originates from the ATP synthase F1 subunit gamma (ATP5C1) gene. It was significantly upregulated in tissues of ossification of the posterior longitudinal ligament, a common orthopedic disease [20]. Hsa_circ_0007292 could promote the progression of ossification of the posterior longitudinal ligament via the miR-508-3p/SATB2 pathway [20]. By analyzing a circRNA microarray from the Gene Expression Omnibus (GEO) database, we found that hsa_circ_0007292 was higher in cartilage tissues of OA patients than normal control. However, the role of hsa_circ_0007292 in OA remains unknown.

High mobility group box-1 (HMGB1) is a member of a damage-associated molecular pattern (DAMP) protein. It stimulates the innate immune system and trigger inflammatory responses through binding to many cell surface receptors, including Toll-like receptors and receptors for advanced glycosylation end products (RAGE) [21]. HMGB1 has been demonstrated to be highly expressed in synovium and synovial fluid of patients with knee OA, and its levels in synovial fluid were related to the severity of synovitis and pain in patients [21]. It was also abnormally expressed in OA joint chondrocytes. In vitro, recombinant HMGB1 protein stimulation increased cytokine production and decreased matrix production in chondrocytes. Intra-articular injection of HMGB1-neutralizing antibodies had cartilage-protective effects in mouse OA models [22]. In this study, we detected the expression profile of hsa_circ_0007292 in OA cartilage tissues and interleukin (IL)-1β-induced chondrocytes, an OA cell model. What’ more, we investigated whether hsa_circ_0007292 functioned in OA by regulating HMGB1 via sponging miRNA.

## Materials and methods

### Bioinformatic analysis

GSE178724 included four patients with OA and four paired controls was downloaded from the GEO database (https://www.ncbi.nlm.nih.gov/gds/). Differentially expressed circRNAs were analyzed by the GEO2R online tool. CircRNAs with *P* < 0.05 and |logFC|> 1 were identified as differentially expressed circRNAs.

The target miRNAs of hsa_circ_0007292 were predicted by circInteractome (https://circinteractome.nia.nih.gov/index.html), StarBase v3.0 (https://starbase.sysu.edu.cn/), and circBank (http://www.circbank.cn/index.html).

### Cartilage tissues

21 OA cartilage tissues were collected from OA patients underwent total knee replacement surgery (12 males and 9 females, age range 56–72 years). Patients with primary knee OA were diagnosed based on the American College of Rheumatology criteria [23] and classified according to the Kellgren-Lawrence scoring system [24]. The inclusion criteria of OA patients were diagnosis of knee OA, aged 50–75 years, Kellgren-Lawrence score ≥ 3, and pain lasting for more than three months. The exclusion criteria of OA patients included secondary OA, recent surgery of the knee, the presence of other rheumatic pathologies, with malignant tumor or autoimmune disease. This study included 5 OA patients with grade 3 and 16 OA patients with grade 4. 17 healthy cartilage samples were obtained from trauma patients who had no history of OA or rheumatoid arthritis (10 males and 7 females, age range 50–67 years). These cartilage tissues were immediately stored in liquid nitrogen after surgery. Written informed consents were obtained from all cartilage donors. This study was approved by the Research Ethics Committee of The Affiliated Hospital of Guangdong Medical University (PJKT2020-006).

### Cell culture and treatment

Two human chondrocytes (CHON-001 and C28/I2) were used in this study. CHON-001 cells were purchased from the American Type Culture Collection (ATCC, Manassas, VA, USA), and C28/I2 cells were from Sigma-Aldrich (Louis, MO, USA). Both cells were maintained in Dulbecco’s modified Eagles’ medium (DMEM; Hyclone, Logan, Utah, USA) supplemented with 10% fetal bovine serum (Gibco, Grand Island, NY, USA), 100 U/mL penicillin and 100 μg/mL streptomycin at 37 °C with 5% CO_2_. IL-1β was used to stimulate chondrocytes to establish OA model in vitro [25, 26]. In this study, chondrocytes were treated with 5, 10, or 20 ng/mL IL-1β for 24 h to construct the OA model.

### Transfection

Three small interfering RNAs (siRNAs) targeting hsa_circ_0007292, miR-1179 mimic, miR-1179 inhibitor and their corresponding controls were synthesized by GenePharma Co., Ltd. (Shanghai, China). The target sequences of hsa_circ_0007292 were as follows: hsa_circ_0007292 siRNA-1 (si#1): 5′-AGCTCTTCACCAGGAGACTAA-3’; hsa_circ_0007292 siRNA-2 (si#2): 5′-CAGCTCTTCACCAGGAGACTA-3’; hsa_circ_0007292 siRNA-3 (si#3): 5’-TGGTGCTGCAGCTCTTCACCA-3’. HMGB1 cDNA was cloned into the pcDNA3.1 plasmid to construct HMGB1 overexpressing plasmid. CHON-001 or C28/I2 cells were transfected with these vectors using Lipofectamine 3000 (Invitrogen, Carlsbad, CA, USA) according to the manufacturer’s instructions.

### Real-time PCR (qRT-PCR) and RNase R treatment

TRIzol reagent (Invitrogen) was utilized to extract total RNA from 38 cartilage tissues (21 OA and 17 healthy control samples) and 2 chondrocyte cell lines. To detect the expression of hsa_circ_0007292 and HMGB1, cDNA was synthesized using M-MLV Reverse Transcriptase (Invitrogen) from 1 μg of total RNA. After preparing PCR reaction systems with SYBR® Green (Promega, Madison, WI, USA), qRT-PCR was performed on ABI 7500 System with GAPDH as an endogenous control. For miRNAs analysis, TaqMan® MicroRNA Reverse Transcription Kit and TaqMan® Universal Master Mix II (Thermo Fisher, Waltham, MA, USA) were used and U6 was used as the endogenous reference. The relative gene expression was calculated by the 2^−ΔΔCt^ method. The primer sequences were listed in Table 1.

**Table 1:** Primer sequences

Gene	Forward primer (5′-3′)	Reverse primer (5′-3′)
hsa_circ_0007292	GCCAAGCTGTCATCACAAAA	TCTCTCAGCTCGGGCATATT
ATP5C1	GGTAGCGGCAGCAAAATATGC	CCACACAGTCCTCGATCTGAG
HMGB1	TATGGCAAAAGCGGACAAGG	CTTCGCAACATCACCAATGGA
GAPDH	CTGGGCTACACTGAGCACC	AAGTGGTCGTTGAGGGCAATG
miR-1179	ACACTCCAGCTGGGAAGCATTCTTTCATT	TGGTGTCGTGGAGTCG
miR-485-3p	ACACTCCAGCTGGGGTCATACACGGCTCTC	TGGTGTCGTGGAGTCG
miR-515-5p	ACACTCCAGCTGGGTTCTCCAAAAGAAAGCAC	TGGTGTCGTGGAGTCG
miR-508-3p	ACACTCCAGCTGGGTGATTGTAGCCTTTTGG	TGGTGTCGTGGAGTCG
U6	CTCGCTTCGGCAGCACA	AACGCTTCACGAATTTGCGT

For RNase R treatment, 2 μg of total RNA from cells were treated with 3 U/μg RNase R (Epicentre Technologies, Madison, WI, USA) for 30 min at 37 °C. The expression of hsa_circ_0007292 and its corresponding linear mRNA ATP5C1 expression was determined by qRT-PCR.

### Cell proliferation analysis

Cell viability was analyzed by CCK-8 assay. Briefly, CHON-001 and C28/I2 cells (3000 cells/well) were seeded into 96-well plates in 100 µL of DMEM medium. After adhering to the plates, cells were transfected with indicated vectors. 48 h after transfection, cells were stimulated with 10 ng/mL IL-1β for 24 h, followed by incubation with CCK-8 reagent (each well adding 10 µL CCK-8; Beyotime, Shanghai, China) for another 1 h. Then, the optical density value was evaluated by a Multiskan microplate reader (Thermo Fisher) at 450 nm.

The proliferation of CHON-001 cells was also evaluated by 5-bromo-12’-deoxyuridine (BrdU). After stimulation with 10 ng/mL IL-1β for 24 h, cells grown on coverslips were incubated with 20 μM BrdU solution for 2 h, fixed with 4% paraformaldehyde for 30 min, and permeabilized with 1 mol/L HCl for 30 min. After blocking with 3% bovine serum albumin at room temperature for 1 h, cells were treated with BrdU monoclonal antibody (BD Biosciences, Franklin Lakes, NJ, USA) overnight at 4 °C. Then, cells were incubated with Alexa FluorR® 594-labeled secondary antibody (Thermo Fisher). Finally, the nucleus was stained with DAPI. A fluorescence microscope was used to capture photos. BrdU positive cell number was calculated in 5 random fields per sample.

### Flow cytometry for cell apoptosis

The apoptosis of chondrocytes was verified by Annexin V-FITC/propidium iodide (PI) Apoptosis Detection Kit (Beyotime). Briefly, after indicated treatment, cells were collected, washed with PBS, and resuspended in binding buffer at a concentration of 1 × 10^6^/mL. Then, cells were incubated with 5 μL AnnexinV-FITC and 5 μL PI for 15 min at room temperature in the dark. Cell apoptosis was analyzed by the BD FACSCalibur flow cytometry (Becton Dickinson, Mountain View, CA).

### ELISA and Western blot

The supernatant of chondrocytes was collected, and the concentrations of TNF‐α, IL‐6 and IL‐8 were detected using specific ELISA kits from Abcam (Cambridge, MA, USA) according to the manufacturers’ instructions.

Proteins from chondrocytes were extracted by lysis buffer containing 1% PMSF. After measuring the concentration with a BCA Protein Assay Kit (Beyotime), an equal amount of denatured protein was separated with SDS-PAGE electrophoresis, transferred onto polyvinylidene difluoride membranes (Millipore, Billerica, MA, USA) and blocked in 5% nonfat-dried milk. Then, the membranes were incubated overnight at 4 °C with primary antibodies against Aggrecan, matrix metallopeptidase (MMP)13 and high mobility group box-1 (HMGB1) (1:1000 dilution; Abcam). After washing three times with TBS-Tween 20 (0.1%), the membranes were incubated with horseradish peroxidase-conjugated secondary antibody at room temperature for 1 h. Protein bands were observed by chemiluminescence imaging using the ECL kit (Beyotime). β-actin was used as an internal control.

### Dual-luciferase reporter assay

The binding sites between hsa_circ_0007292 and miR-1179 were predicted by the circInteractome. According to their binding sites, hsa_circ_0007292 wild type (circ_0007292-Wt) and mutant (circ_0007292-Mut) reporter vectors were constructed by cloning into the pGL3 luciferase vector. These plasmids and miR-1179 mimic were then co-transfected into CHON-001 cells using Lipofectamine 3000. A Dual Luciferase Reporter Assay (Promega, Madison, WI, USA) was carried out to analyze the luciferase activities 48 h post-transfection.

### RNA pull-down

Biotinylated miR-1179 probe and biotin-labeled mock probe were obtained from Genepharma Co., Ltd. C28/I2 cells were transfected with 100 nM Biotin-labeled probes for 48 h. Cells were lysed by RIP buffer containing protease inhibitors and RNase inhibitor. To generate probe-coated beads, the cell lysates were incubated with M-280 streptavidin magnetic beads (Invitrogen) overnight at 4 °C. Then, RNA complex bound to the beads were eluted and extracted by Trizol. Lastly, hsa_circ_0007292 and HMGB1 enrichment pulled down by miR-1179 probe were measured by qRT-PCR.

### Statistical analysis

The data are shown as the mean ± standard deviation (SD). All analyses were performed using GraphPad Prism 8.0 software (GraphPad, Inc.). The correlations among hsa_circ_0007292, miR-1179, and HMGB1 were analyzed by Pearson’s correlation analysis. The significance of difference was detected by Student’s t test or one-way ANOVA. *P* < 0.05 was considered statistically significant.

## Results

### Hsa_circ_0007292 is elevated in OA tissues and IL-1β-induced chondrocytes

**Fig. 1:**
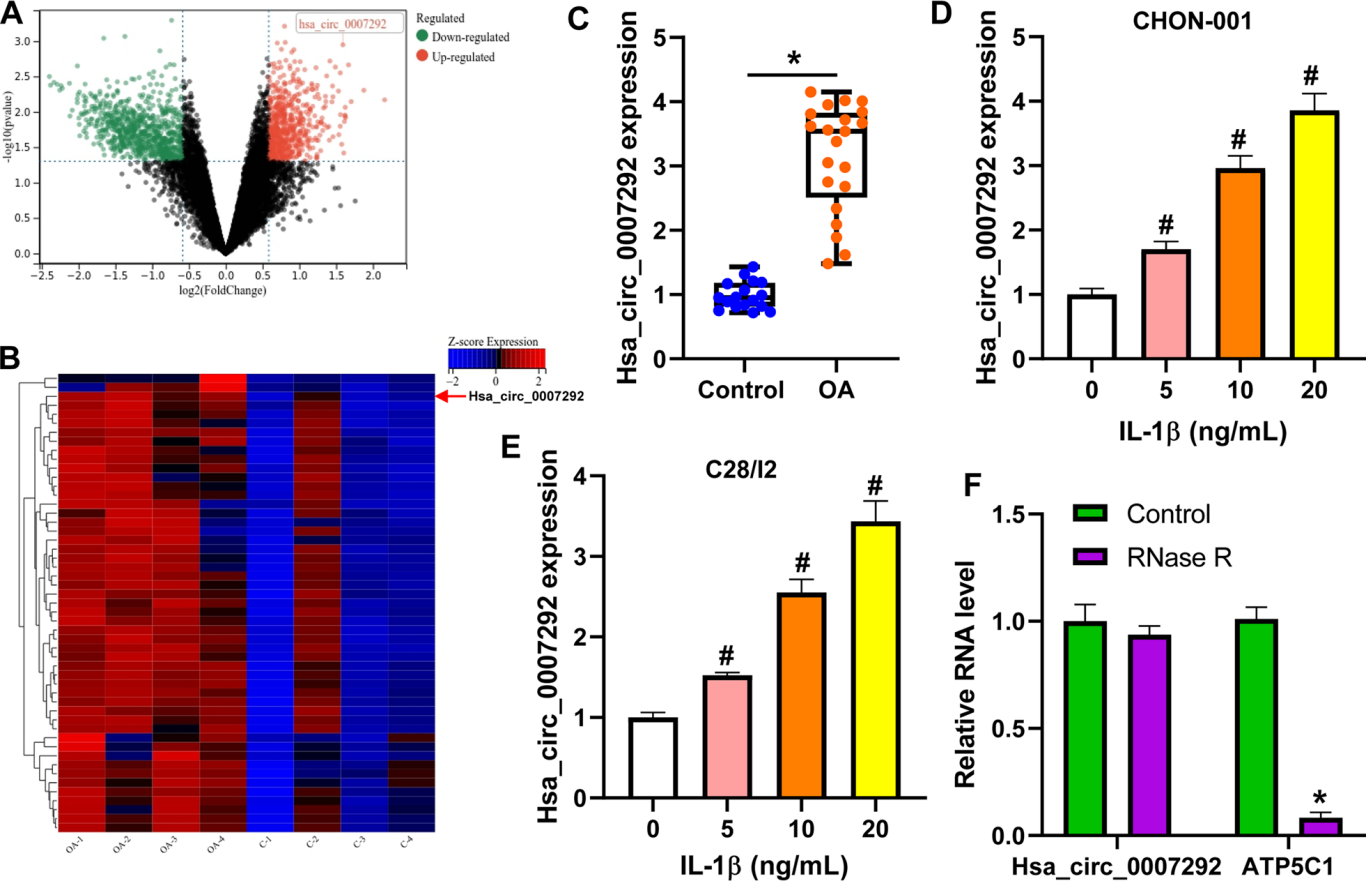
Hsa_circ_0007292 is elevated in OA tissues and IL-1β-induced chondrocytes. **A**: A volcano plot illustrates the differentially expressed circRNAs. **B**: A heatmap representing the top 50 upregulated circRNAs in cartilage tissues of OA patients and controls. **C**: Hsa_circ_0007292 expression was assayed by qRT-PCR in 21 OA tissues and 17 healthy controls. The expression of hsa_circ_0007292 was increased in CHON-001 (**D**) and C28/I2 (**E**) cells under IL-1β treatment. **F**: qRT-PCR was used to detect the expression of hsa_circ_0007292 and ATP5C1 in 10 ng/mL IL-1β-stimulated CHON-001 cells treated with or without RNase R. ^*^P < 0.05 vs. control; ^#^*P* < 0.05 vs. 0 ng/mL

From the GSE178724 datasets, we found 622 differentially expressed circRNAs, including 131 upregulated circRNAs and 491 downregulated circRNAs, which was shown by a volcano plot (Fig. 1A). A heatmap of the top 50 upregulated circRNAs was presented in Fig. 1B, among which hsa_circ_0007292 was one of the most upregulated circRNAs. Hence, we collected 21 cartilage tissues from OA patients and 17 normal cartilage samples to further analyze the expression of hsa_circ_0007292 by qRT-PCR. The results showed that compared with normal cartilage tissues, hsa_circ_0007292 expression was significantly increased in cartilage tissues of OA patients (Fig. 1C). Additionally, we found that IL-1β stimulation dramatically promoted the expression of hsa_circ_0007292 in both human chondrocytes (CHON-001 and C28/I2) in a dose-dependent manner (Fig. 1D and E). Hsa_circ_0007292 was resistant to RNase R digestion, while its corresponding linear mRNA ATP5C1 was reduced after RNase R treatment (Fig. 1F). These data indicated that hsa_circ_0007292 expression was elevated in OA.

### Silencing of Hsa_circ_0007292 alleviates IL-1β-induced chondrocyte injury

**Fig. 2:**
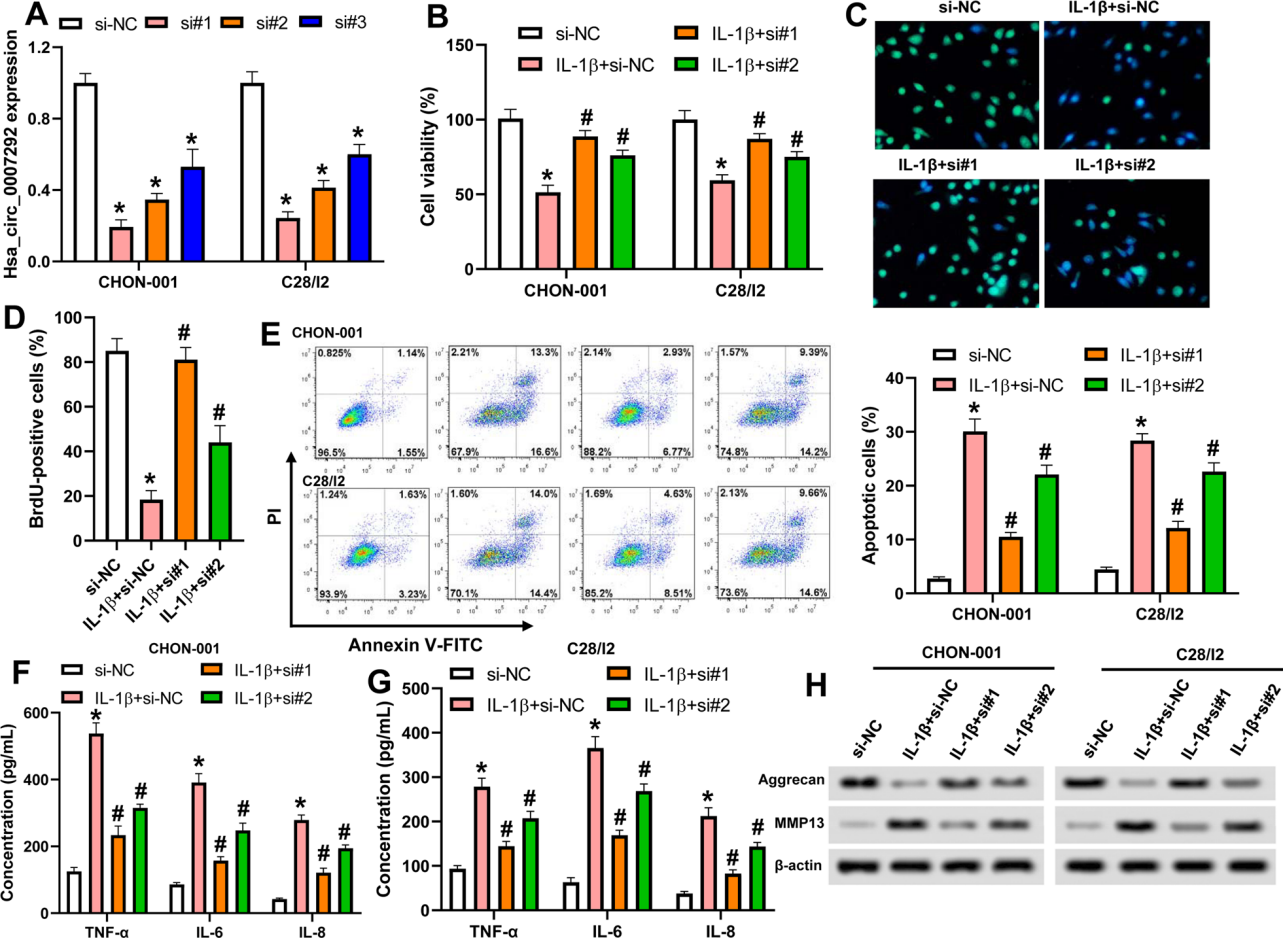
Silencing of hsa_circ_0007292 alleviates IL-1β-induced chondrocyte injury. CHON-001 and C28/I2 cells were stimulated with 10 ng/mL IL-1β and transfected with indicated vectors. **A**: Hsa_circ_0007292 expression was analyzed. **B**: The viability of CHON-001 and C28/I2 cells was determined by CCK-8. **C** and **D**: The proliferation of CHON-001 cells was detected by BrdU and BrdU-positive cells was calculated. **E**: Flow cytometry assay for the apoptosis of chondrocytes. **F** and **G**: The concentrations of TNF‐α, IL‐6, and IL‐8 in the culture medium of CHON-001 and C28/I2 cells were measured by ELISA. **H**: The expression of Aggrecan and MMP13 was examined by western blot. ^*^P < 0.05 vs. si-NC; ^#^*P* < 0.05 vs. IL-1β + si-NC. si#1: Hsa_circ_0007292 siRNA-1; si#2: Hsa_circ_0007292 siRNA-2

To investigate the role of hsa_circ_0007292 in OA, three siRNAs were transfected into CHON-001 and C28/I2 cells. qRT-PCR assay revealed that hsa_circ_0007292 was successfully downregulated by these siRNAs (Fig. 2A). CCK-8 method showed that IL-1β dramatically decreased cell viability, and compared with IL-1β + si-NC group, knockdown of hsa_circ_0007292 notably increased the viability of both chondrocytes (Fig. 2B). Similarly, BrdU assay demonstrated that silencing of hsa_circ_0007292 elevated the proliferation of CHON-001 cells (Fig. 2C and D). Meanwhile, knockdown of hsa_circ_0007292 suppressed IL-1β-induced apoptosis, and the secretion of inflammatory factors, including TNF‐α, IL‐6, and IL‐8 in both chondrocytes (Fig. 2E–G). Additionally, compared with IL-1β + si-NC group, downregulation of hsa_circ_0007292 facilitated Aggrecan expression, while inhibited the expression of MMP13 (Fig. 2H). These results indicated that downregulation of hsa_circ_0007292 alleviated IL-1β-induced apoptosis, extracellular matrix degradation and inflammatory response in chondrocytes.

### Hsa_circ_0007292 sponges miR-1179

**Fig. 3:**
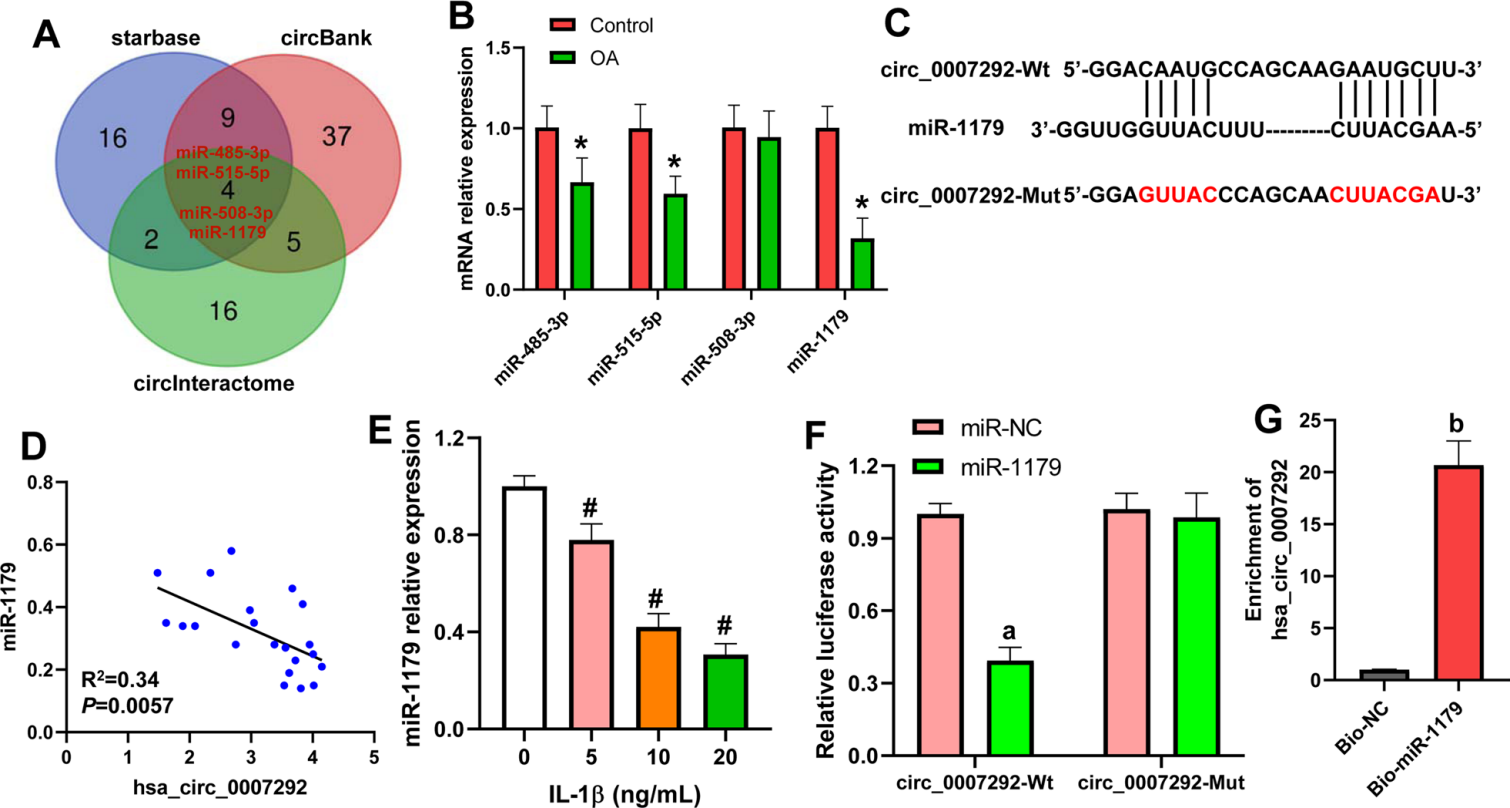
Hsa_circ_0007292 sponges miR-1179. **A**: Venn diagram of the overlapping miRNAs targeted hsa_circ_0007292. **B**: The expression of miR-485-3p, miR-515-5p, miR-508-3p, and miR-1179 was detected in 21 OA tissues and 17 controls. **C**: The predicted binding sites between hsa_circ_0007292 and miR-1179. **D**: The correlation between hsa_circ_0007292 and miR-1179 in OA tissues. **E**: miR-1179 expression in CHON-001 cells after treatment with different concentrations (0, 5, 10 and 20 ng/mL) of IL-1β. **F**: Dual-luciferase reporter assay. G: RNA pull-down assay showed that the enrichment of hsa_circ_0007292 was elevated in Bio-miR-1179 compared with Bio-NC. ^*^P < 0.05 vs. Control; ^#^*P* < 0.05 vs. 0 ng/mL; ^a^*P* < 0.05 vs. miR-NC; ^b^*P* < 0.05 vs. Bio-NC

Previous study has demonstrated that hsa_circ_0007292 was expressed in the cytoplasm, and it functioned as a sponge of miRNA [20]. Through three bioinformatics prediction tools, including circInteractome, StarBase v3.0, and circBank, we found 4 miRNAs (miR-485-3p, miR-515-5p, miR-508-3p and miR-1179) that bound to hsa_circ_0007292 (Fig. 3A). The expression of these miRNAs was confirmed in OA tissues by qRT-PCR. The results showed that compared with the control, the expression of miR-485-3p, miR-515-5p and miR-1179 was significantly decreased in OA tissues, with the lowest of miR-1179 (Fig. 3B). Hence, we further investigated the relationship between hsa_circ_0007292 and miR-1179. The binding sites between them were shown in Fig. 3C. There was a negative correlation between them in the cartilage tissues from OA patients (Fig. 3D). Meanwhile, miR-1179 expression was notably inhibited by IL-1β in chondrocytes (Fig. 3E). Dual-luciferase reporter assay and RNA pull-down assay was used to confirm the direct binding between hsa_circ_0007292 and miR-1179. As shown in Fig. 3F, miR-1179 mimic significantly reduced the luciferase activity of chondrocytes transfected with hsa_circ_0007292-Wt, but did not affect that with hsa_circ_0007292-Mut. Compared with Bio-NC, a significant enrichment of hsa_circ_0007292 was observed by Bio-miR-1179 in chondrocytes (Fig. 3G). Taken together, these data suggested that hsa_circ_0007292 was a sponge of miR-1179 in chondrocytes.

### miR-1179 relieves IL-1β-induced chondrocyte injury via targeting HMGB1

**Fig. 4:**
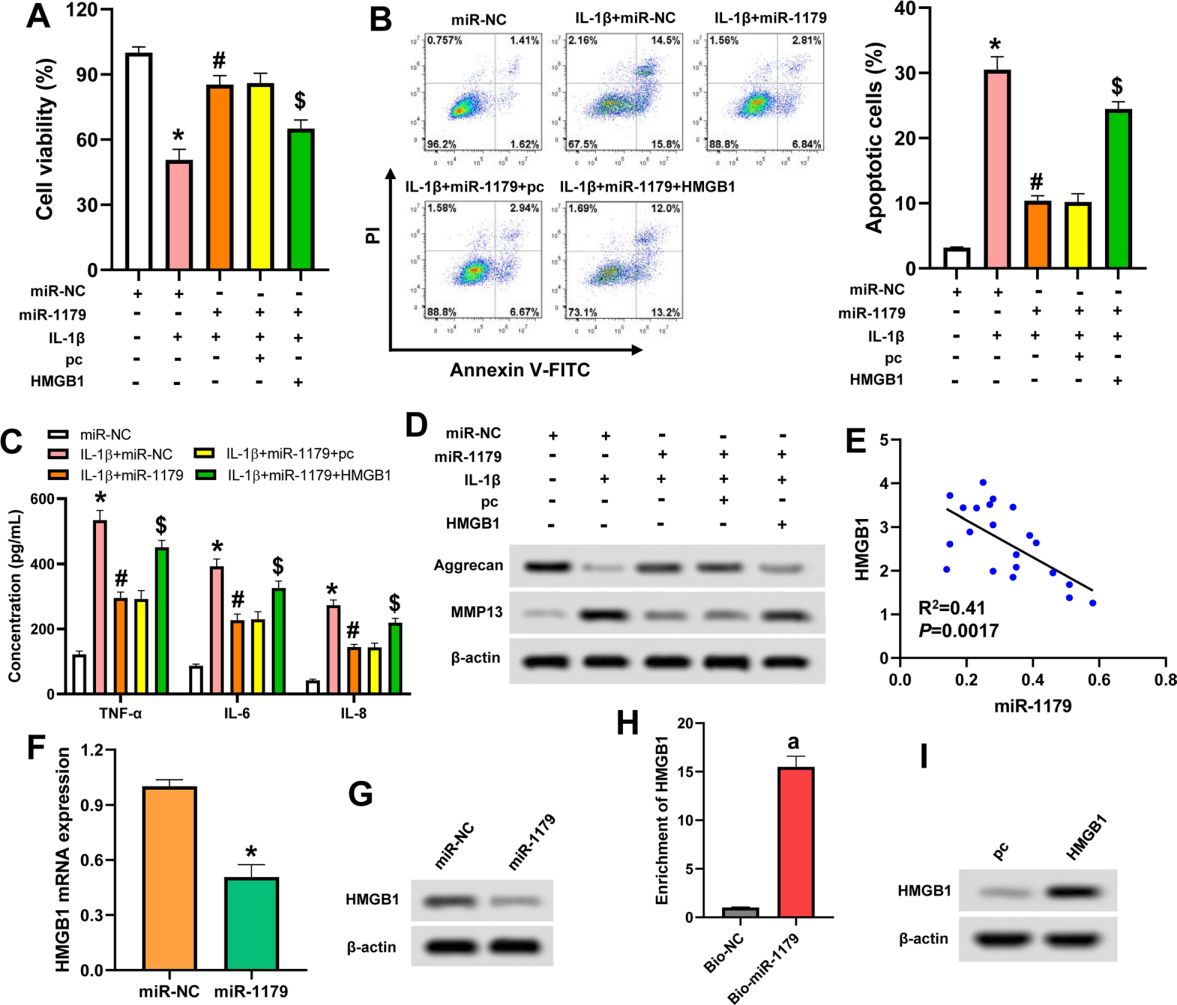
miR-1179 relieves IL-1β-induced chondrocyte injury via targeting HMGB1. **A**: CHON-001 cells were transfected with designated vectors and stimulated with or without 10 ng/mL IL-1β for 24 h. Cell viability was analyzed by CCK-8 assay. **B**: The apoptosis of CHON-001 cells was detected by flow cytometry. **C**: ELISA assay for the secretion of TNF‐α, IL‐6, and IL‐8. **D**: Western blot was utilized to measure the protein levels of Aggrecan and MMP13. **E**: A negative correlation was found between the expression of miR-1179 and HMGB1 in 21 OA tissues. **F**: qRT-PCR analysis for HMGB1 mRNA expression. **G**: HMGB1 protein level was decreased by miR-1179. **H**: RNA pull-down assay showed that the enrichment of HMGB1 was elevated in Bio-miR-1179 compared with Bio-NC. **I**: HMGB1 protein expression was significantly increased in CHON-001 cells after transfecting with pcDNA3.1-HMGB1 plasmid. ^*^P < 0.05 vs. miR-NC; ^#^*P* < 0.05 vs. IL-1β + miR-NC; ^$^*P* < 0.05 vs. IL-1β + miR-1179; ^a^*P* < 0.05 vs. Bio-NC

The effect of miR-1179 on IL-1β-induced chondrocyte injury was evaluated. As shown in Fig. 4A–D, miR-1179 mimic significantly increased cell proliferation, inhibited apoptosis and the secretion of inflammatory factors (TNF‐α, IL‐6, and IL‐8), elevated Aggrecan expression, and reduced the expression of MMP13. These results demonstrated that miR-1179 could relieve IL-1β-induced chondrocyte injury.

HMGB1 has been found to be highly expressed, and it promoted chondrocyte apoptosis and inflammation in OA [27]. Li et al*.* [28] demonstrated that HMGB1 was a target of miR-1179 in gastric cancer. Therefore, we further analyzed whether miR-1179 functioned in OA via targeting HMGB1. A negative correlation between miR-1179 and HMGB1 was found in our collected OA tissues (Fig. 4E). miR-1179 inhibited the expression of HMGB1 both at transcription and translation levels (Fig. 4F and G). The enrichment of HMGB1 on Bio-miR-1179 was higher than Bio-NC (Fig. 4H). These results indicated that HMGB1 was a target of miR-1179 in chondrocytes. Additionally, upregulation of HMGB1 dramatically attenuated the influences of miR-1179 on the proliferation, apoptosis, inflammation and expression of Aggrecan and MMP13 (Fig. 4A–D, I). This demonstrated that miR-1179 relieved IL-1β-induced chondrocyte injury via targeting HMGB1.

### Hsa_circ_0007292 regulates HMGB1 expression via miR-1179

**Fig. 5:**
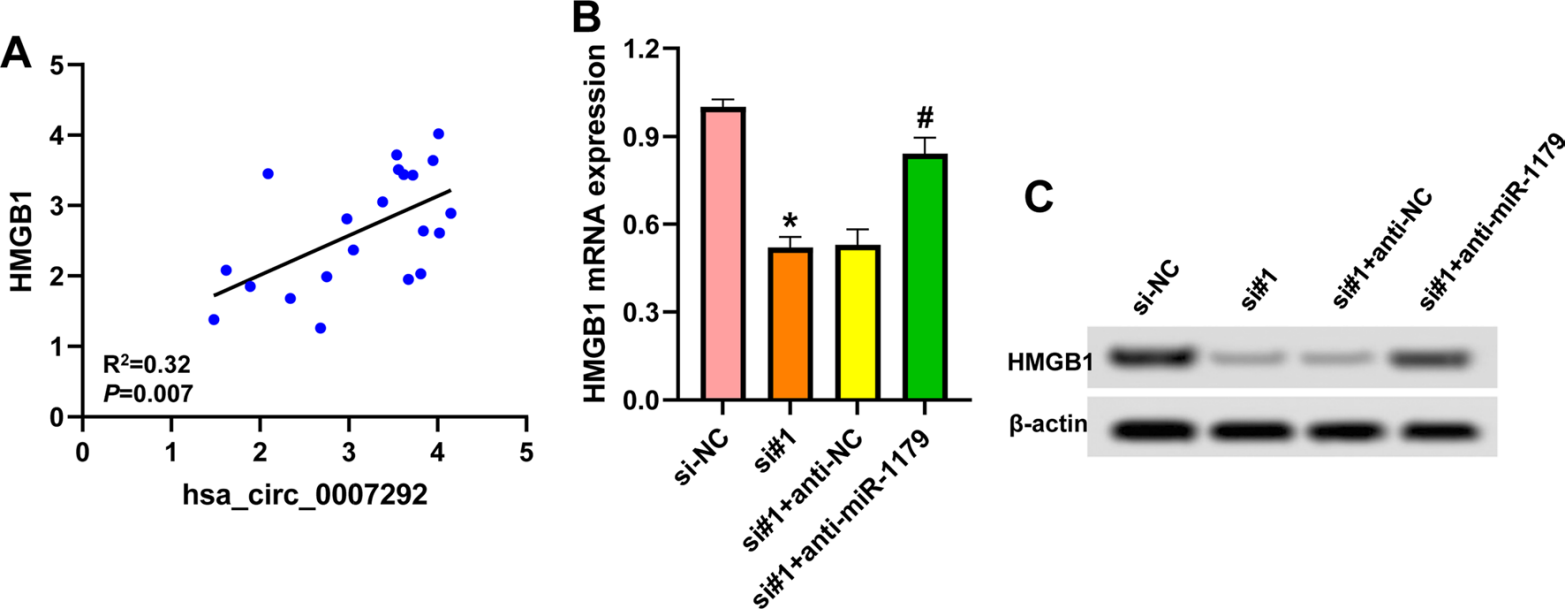
Hsa_circ_0007292 regulates HMGB1 expression via miR-1179. **A**: The correlation between hsa_circ_0007292 and HMGB1 in 21 OA tissues. **B**: CHON-001 cells were transfected with hsa_circ_0007292 siRNA (si#1)/si-NC or co-transfected with hsa_circ_0007292 siRNA and miR-1179 inhibitor (anti-miR-1179)/control (anti-NC) for 48 h, followed by stimulation with 10 ng/mL IL-1β for 24 h. HMGB1 mRNA expression was detected by qRT-PCR. **C**: Western blot was used to measure HMGB1 protein level. ^*^P < 0.05 vs. si-NC; ^#^*P* < 0.05 vs. si#1

Next, we explored whether hsa_circ_0007292 could affect the expression of HMGB1. A positive correlation between hsa_circ_0007292 and HMGB1 was found in OA tissues (Fig. 5A). In IL-1β-treated CHON-001 cells, both the mRNA and protein levels of HMGB1 were reduced after transfection with hsa_circ_0007292 siRNA (Fig. 5B and C). Meanwhile, we also found miR-1179 inhibitor notably reversed the effects of hsa_circ_0007292 silencing on HMGB1 expression (Fig. 5B and C). These data indicated that hsa_circ_0007292 positively regulated HMGB1 expression via miR-1179.

### Hsa_circ_0007292 functions via regulating HMGB1 in OA

**Fig. 6:**
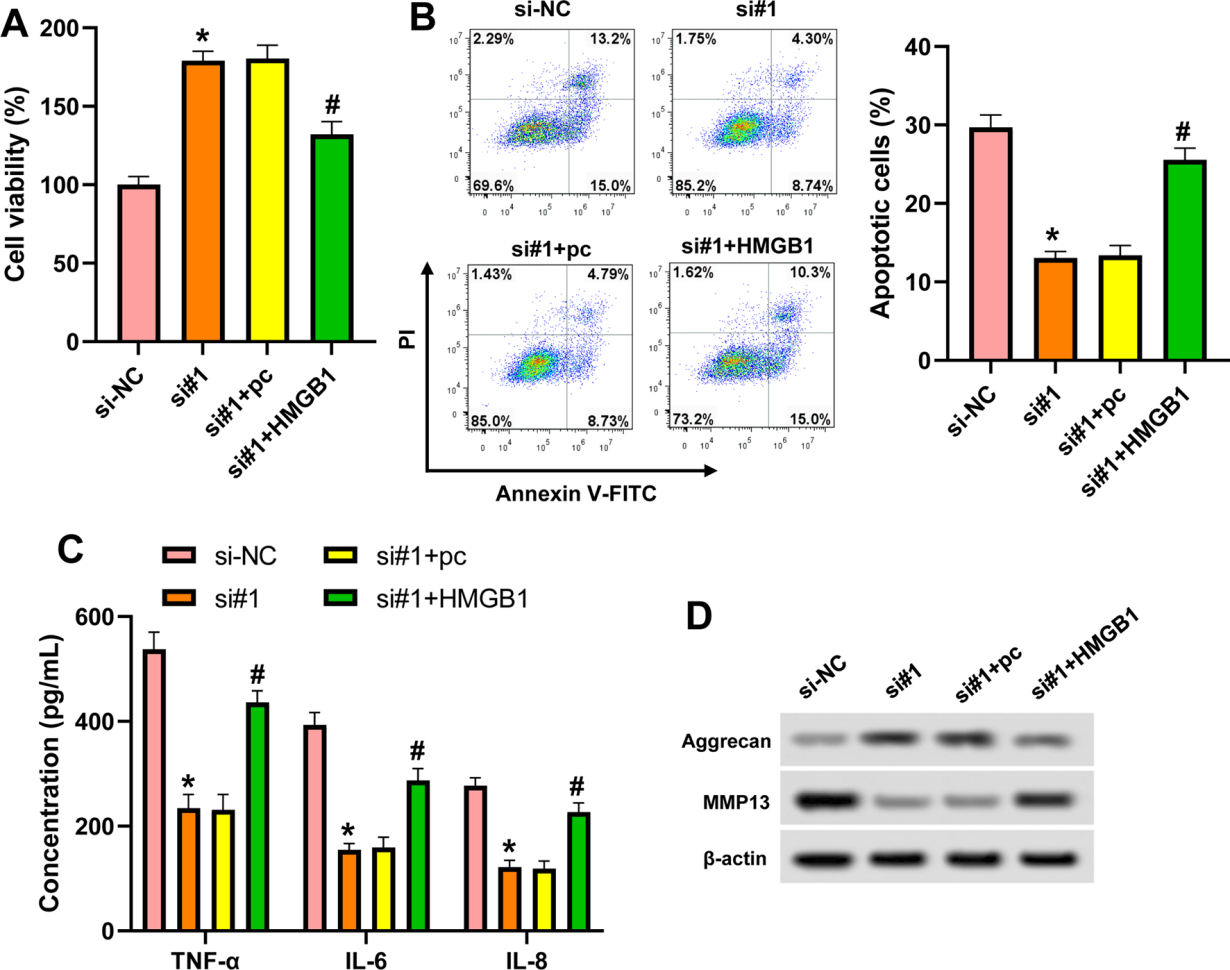
Hsa_circ_0007292 functions via positively regulating HMGB1 in OA. CHON-001 cells were transfected with hsa_circ_0007292 siRNA (si#1)/si-NC or co-transfected with hsa_circ_0007292 siRNA and HMGB1 overexpressing plasmid/control (pc) for 48 h, followed by stimulation with 10 ng/mL IL-1β for 24 h. **A**: Cell viability was measured by CCK-8. **B**: Flow cytometry assay for cell apoptosis. **C**: The secretion of TNF‐α, IL‐6, and IL‐8 was detected by ELISA. **D**: The protein levels of Aggrecan and MMP13 were determined by western blot. ^*^P < 0.05 vs. si-NC; ^#^*P* < 0.05 vs. si#1

To further confirm whether hsa_circ_0007292 functioned in OA via regulating HMGB1, a rescue experiment was performed using HMGB1-overexpressing plasmid. As revealed in Fig. 6A–D, compared with hsa_circ_0007292 siRNA-treated cells, the combination of hsa_circ_0007292 siRNA and HMGB1 upregulation significantly reduced proliferation, promoted apoptosis and the secretion of TNF‐α, IL‐6 and IL‐8, reduced Aggrecan expression, and increased MMP13 level. These results suggested that silencing of hsa_circ_0007292 alleviated IL-1β-induced chondrocyte injury through the regulation of HMGB1.

## Discussion

A lot of researchers have found that many aberrant expressions of genes in OA articular chondrocytes contribute to the pathogenesis of OA, the detailed regulator mechanism of OA remains to be elucidated. Noncoding RNAs, which mainly include miRNAs, long noncoding RNA (lncRNAs) and circRNAs, are important RNA molecules that regulate a variety of biological and pathological processes [29–34]. Studies have demonstrated that noncoding RNAs function in musculoskeletal conditions [35–38]. Increasing evidence showed that circRNAs played critical roles in the occurrence and development of OA. For example, Zhou et al*.* [14] demonstrated that the upregulation of circRNA.33186 contributes to the pathogenesis of OA. CircRERE expression was suppressed in OA cartilage and chondrocytes. It exerted chondroprotective effects by inhibiting apoptosis, promoting proliferation, and affecting anabolic and catabolic biomarker synthesis in OA [39]. Compared with control, the expression of circSCAPER was higher in OA cartilage tissues and IL-1β-treated chondrocytes. Downregulation of circSCAPER suppressed IL-1β-induced ECM degradation and proliferation arrest, while relieved IL-1β-evoked apoptosis in chondrocytes, suggesting that circSCAPER was responsible for the development of OA [40]. In this study, we found that hsa_circ_0007292 was a highly expressed circRNAs in OA cartilage tissues through sequencing data. Furthermore, we also reported that hsa_circ_0007292 was increased in our collected OA cartilage tissues and IL-1β-induced chondrocytes. This indicated that hsa_circ_0007292 might play an important role in the pathogenesis of OA.

Chondrocyte apoptosis and the decline of chondrocyte survival are responsible for articular cartilage degradation in OA [41]. Inflammation and ECM degradation are considered to be critical reasons to accelerate OA progression [42]. Aggrecan is one major proteoglycan of articular cartilage and it provides cartilage with the ability to withstand the compressive loads [43]. MMP13 is a critical cartilage-degrading enzyme that degrade ECM molecules in OA pathogenesis [6]. In this study, we found that downregulation of hsa_circ_0007292 facilitated proliferation, inhibited apoptosis and inflammation, increased Aggrecan expression, decreased the expression of MMP13 in IL-1β-induced chondrocytes. These results indicated that hsa_circ_0007292 was involved in the progression of OA and it might be a therapeutic target.

Studies have found that circRNAs could act as miRNA sponges, leading to the repression of miRNA function [44, 45]. Importantly, Jiang et al*.* [20] has demonstrated that hsa_circ_0007292 was predominantly expressed in the cytoplasm. Hence, we investigated whether hsa_circ_0007292 functioned in OA via acting as miRNA sponges. Bioinformatic analysis revealed that hsa_circ_0007292 might serve as a sponge of miR-1179. Dual-luciferase reporter assay and RNA pull-down assay confirmed the direct binding between hsa_circ_0007292 and miR-1179. The role of miR-1179 has not been demonstrated in OA. In our study, we found that miR-1179 was under-expressed in OA cartilage tissues and IL-1β-induced chondrocytes. miR-1179 could increase proliferation, inhibit apoptosis and inflammation, and suppress ECM degradation in IL-1β-induced chondrocytes. This indicated that miR-1179 could relieve IL-1β-induced chondrocyte injury in OA. There was a negative correlation between hsa_circ_0007292 and miR-1179 in OA tissues. These data suggested that hsa_circ_0007292 might function as a sponge of miR-1179 in OA.

We also explored the target mRNA of miR-1179 in OA. Previous study has verified that HMGB1 was a target of miR-1179 in gastric cancer [28]. Studies have demonstrated that HMGB1 was highly expressed in synovium, synovial fluid and joint chondrocytes of patients with knee OA and it promoted the progression of OA [21, 22]. Therefore, we further evaluated whether HMGB1 was a target of miR-1179 in OA. In this study, there was a negative correlation between miR-1179 and HMGB1 in OA tissues. miR-1179 significantly suppressed HMGB1 expression in chondrocytes. An RNA pull-down assay further confirmed the direct binding between miR-1179 and HMGB1 in chondrocytes. This indicated the HMGB1 was a target of miR-1179 in OA. As expected, HMGB1 overexpression could reverse the effects of miR-1179 on the proliferation, apoptosis, inflammation and ECM biomarkers in IL-1β-induced chondrocytes. These results suggested that miR-1179 targeted HMGB1 in OA.

Additionally, the relationship between hsa_circ_0007292 and HMGB1 was further evaluated. There was a positive correlation between hsa_circ_0007292 and HMGB1 in OA tissues. Downregulation of hsa_circ_0007292 led to the suppression of HMGB, the effect of which was attenuated by miR-1179 inhibitor. And HMGB1 overexpression could reverse the influences of hsa_circ_0007292 downregulation on IL-1β-induced chondrocyte injury. These data indicated that hsa_circ_0007292 functioned in OA chondrocytes by positively regulating HMGB1 through acting as a sponge of miR-1179.

## Conclusions

Our research found a new circRNA contributes to OA. We found that hsa_circ_0007292 was highly expressed in the cartilage tissues of OA, and it could induce apoptosis, inflammation and ECM degradation in OA chondrocytes. Mechanistically, hsa_circ_0007292 acted as a sponge of miR-1179, and further increased HMGB1 expression. This study indicated that downregulation of hsa_circ_0007292 might be a promising method to treat OA.

## Data Availability

The datasets generated and analysed during the current study are available from the corresponding author on reasonable request.
